# Urinary phthalate metabolites in relation to serum anti-Müllerian hormone and inhibin B levels among women from a fertility center: a retrospective analysis

**DOI:** 10.1186/s12978-018-0469-8

**Published:** 2018-02-23

**Authors:** Yao-Yao Du, Na Guo, Yi-Xin Wang, Xiang Hua, Tao-Ran Deng, Xue-Mei Teng, Yang-Cheng Yao, Yu-Feng Li

**Affiliations:** 1 Reproductive Medicine Center, Tongji Hospital, Tongji Medical College, Huazhong University of Science and Technology, 1095 JieFang Avenue, Wuhan, Hubei People’s Republic of China; 2 Department of Occupational and Environmental Health, School of Public Health, Tongji Medical College, Huazhong University of Science and Technology, Wuhan, Hubei People’s Republic of China; 3 Key Laboratory of Environment and Health, Ministry of Education & Ministry of Environmental Protection, and State Key Laboratory of Environmental health (incubating), School of Public Health, Tongji Medical College, Huazhong University of Science and Technology, Wuhan, Hubei People’s Republic of China

**Keywords:** Phthalates, Anti-Müllerian hormone, Inhibin B, Ovarian reserve, Endocrine disruptors

## Abstract

**Background:**

Phthalates, a class of endocrine disruptors, have been demonstrated to accelerate loss of ovarian follicle pool via disrupting folliculogenesis, and lead to diminished ovarian reserve. However, human data are limited. Here, we aimed to examine whether urinary phthalate metabolites are correlated with markers of ovarian reserve among women attending a fertility clinic.

**Methods:**

We measured eight phthalate metabolites in urine samples collected from 415 women seeking infertility treatment at the Reproductive Medicine Center of Tongji Hospital, Wuhan, China. Data on measures of ovarian reserve, as indicated by serum anti-Müllerian hormone (AMH) and inhibin B (INHB) levels, were retrieved retrospectively through electronic medical charts. Multivariate linear models were performed to estimate the associations of urinary phthalate metabolites and serum AMH and INHB. We further explored the potential nonlinearity of the relationships with restricted cubic spline analysis.

**Results:**

Overall, we found largely null associations between urinary phthalate metabolites and serum AMH. The multivariable adjusted differences in serum INHB levels comparing the highest quartile of urinary MEHP to the lowest were − 18.29% (95% CI: − 31.89%, − 1.98%; *P-*trend = 0.04). Women in the second to fourth quartiles of MEOHP had a significant decrease of − 23.74% (95% CI: −35.85%, − 9.24%), − 19.91% (95% CI: −33.30%, − 3.82%) and − 20.23% (95% CI: −34.43%, − 2.96%), respectively, in INHB levels compared to the first quartile. In the spline analysis, we identified a nonlinear relationship between MEOHP exposure and serum INHB.

**Conclusions:**

We provided evidence for a negative association between urinary concentrations of certain phthalate metabolites and serum INHB levels, suggesting an adverse effect of phthalates exposure on growing antral follicles. Whether phthalates exposure at environmentally level will pose a risk for ovarian reserve needs further investigation.

**Electronic supplementary material:**

The online version of this article (10.1186/s12978-018-0469-8) contains supplementary material, which is available to authorized users.

## Plain English summary

Ovarian reserve is reflected by the resting follicle pool. Accelerated depletion of ovarian follicles, manifested as increased rate of ovarian aging, has serious consequences. It will not only lead to reduced fertility but also to non-reproductive health problems through early menopause. Phthalates are a group of synthetic industrial chemicals. Environmental exposure to phthalates in the general population is ubiquitous, and has aroused growing public health concern based on their endocrine disrupting potency. Evidence from toxicological studies has suggested a role for phthalates in accelerating loss of ovarian follicle pool via disrupting follicular development, and consequently leading to diminished ovarian reserve. However, human data are limited, and whether they can execute comparable effects in humans needs to be elucidated. Therefore, in this retrospective study, we measured eight phthalate metabolites in urine samples collected from 415 women attending a fertility clinic in China, and examined whether phthalate exposure is correlated with serum anti-Müllerian hormone (AMH) and inhibin B (INHB) levels—two well-established markers of ovarian reserve. Our study provided evidence for a negative association between urinary concentrations of certain phthalate metabolites and serum INHB levels, suggesting an adverse effect of phthalate exposure on growing antral follicles. Moreover, we identified a similar decrease in INHB after a threshold level of select phthalates was met, and younger women had larger decrease in INHB levels compared to those of advanced age. However, we found largely null associations between urinary phthalate metabolites and serum AMH. Whether phthalate exposure at environmentally level will pose a risk for ovarian reserve needs further investigation.

## Background

Ovarian reserve is established early in life with a finite number of oocytes. It gradually declines across the reproductive lifespan of a woman until the menopause, paralleled with a reduction in oocyte quality [1]. Apart from age, there are other factors contributing to the depletion of ovarian reserve, including genetic factors, iatrogenic causes, and autoimmune diseases. Recent research has raised concerns that environmental exposure may pose a risk for ovarian function, leading to reduced fertility, premature ovarian failure, and even long-term health problems through early onset of menopause [2]. Among the toxicants of concern is one class of the endocrine disrupting chemicals (EDCs)—phthalates, which have been linked to a broad range of adverse reproductive outcomes in epidemiological and toxicological studies [3].

Phthalates are produced in high volume worldwide and used in the synthesis of polyvinyl chloride products, for example, building supplies, food packaging, and medical devices, to impart flexibility and durability. They are also applied in the formation of cosmetics, personal care products and the coatings of medications, as solvents or excipients [4]. Importantly, they have the potential to leach out from these materials during transportation and storage, and enter the body through ingestion, dermal absorption and inhalation. ‘Every day’ exposure to phthalates, at home or in workplace, is ubiquitous corroborated by the fact that many phthalates or their metabolites are found at measurable concentrations in the biological fluids within most of the general population [5–7].

In recent years, accumulated data from experimental studies has demonstrated that certain phthalates have the potential to accelerate loss of ovarian follicle pool via disrupting folliculogenesis at different stages [8]. Di(2-ethylhexyl) phthalate (DEHP) has been shown to deplete the pool of primordial follicles either by disrupting primordial follicle assembly via dysregulated gene expression or by accelerating initial follicular recruitment through altered PI3K signaling [9, 10]. In vivo or in vitro exposed to DEHP, di-n-butyl phthalate (DBP) or their monoesters increased atresia of antral follicles through mechanisms of decreased estradiol (E_2_) production, elevated apoptosis gene expression or oxidative stress damage [11–13]. In animal models, gestational exposure to mono(2-ethylhexyl) phthalate (MEHP), a metabolite of DEHP, resulted in premature ovarian aging in female offspring manifested as shortened reproductive lifespan, which may be a result of increased rate of follicle recruitment and maturation [14]. As the ovarian reserve reflects primarily the resting pool of primordial follicles, together with follicles recruited into the later preantral and antral stages, reduction in the number of follicles at any stage may lead to reduced ovarian reserve [1]. Notably, in our previous study, phthalates have been detected in the follicular fluid of women seeking infertility treatment [15], a population at higher reproductive risk due to the possibility that they may have the highest exposures or more vulnerable to phthalate-induced toxicity. Thus, it is reasonable to hypothesize that phthalates may be capable of interfering the folliculogenesis process, and result in diminished ovarian reserve.

Nevertheless, although the results from basic research are consistent enough to imply a relationship between phthalate exposure and impaired folliculogenesis, the data from clinical studies are scarce. An earlier study has reported a significant decrease in antral follicle count (AFC) toward higher levels of DEHP metabolites in women seeking infertility treatment [16]. While AFC is a routine biomarker of ovarian reserve in clinical practice, its inherent limitations derived from difficulties in standardized measurement and incapability of reflecting the health status of follicles, for example, inclusion of atretic follicles, suggest that a more accurate and robust indicator would be needed [17, 18]. Produced predominantly by preantral and small antral follicles, anti-Müllerian hormone (AMH) acts as a regulator of follicular recruitment [18]. Interest in AMH’s role in reproduction has heightened of late. Emerging data have shown that, with the development of fully automated assays, AMH becomes a substantially better indicator of ovarian reserve than AFC, not only because of its objectivity and potential standardization of analysis but also its advantage of reflecting both the quantity and quality aspects of the follicle pool [19]. Inhibin B (INHB), secreted by granulosa cells of small growing follicles, is also a biomarker of ovarian aging. With its ability to suppress the rise in follicle-stimulating hormone (FSH), INHB is thought to represent the early event in reproductive senescense [20]. Therefore, in the present study, we aimed to examine whether urinary phthalate metabolites are correlated with diminished ovarian reserve (DOR), as indicated by serum AMH and INHB levels among women attending a fertility clinic.

## Methods

### Study participants

This retrospective cohort study was conducted at the Reproductive Medicine Center of Tongji Hospital, Wuhan, China between November 2016 and December 2016. We recruited participants based on the following criteria: 1) women aged between 20 and 45 years, with indications for in vitro fertilization (IVF) or intracytoplasmic sperm injection (ICSI); 2) women who provided one urine sample and signed an informed consent on the day of ovum pick-up; 3) women who had a serum hormone measurement at the first consultation and the tests were within the 4-month period before urine collection. Women who had an oophorectomy (*n* = 2) or gonadotoxic therapy, or with chromosomal abnormality (n = 2), or had oral contraceptive use in the last three months before hormone measurement were excluded from the study, leaving 415 women eligible for participation. At enrollment, the participants completed detailed questionnaires on their demographics, medical history, diets, smoking habits, and other lifestyle factors under the guidance of an investigator. Approval of the study was obtained from the Institutional Review Board of Tongji Hospital.

### Hormone measurement and clinical data

All women had serum AMH, INHB, and AFC measured as a routine fertility assessment at the clinic. Serum AMH was determined at the initial visit of the clinic. Serum INHB and AFC were assessed between day 2 and 4 of a menstrual cycle prior to stimulation. The AMH and INHB assays were conducted using the enzyme-linked immunuosorbent assay (ELISA) kit (Ansh Labs, Webster, TX, USA) based on the automated DS2 (Dynex Technologies, Chantilly, USA) ELISA processing system. For both AMH and INHB assays, six standards and two quality controls of high and low concentrations were run with serum samples in each assay to monitor accuracy and precision. The standard calibration curves were linear (*r*^2^ > 0.99) with a measuring range of 0.06–18 ng/mL for AMH, and a range of 10–1500 pg/mL for INHB. The relative standard deviations (RSD) of both assays were less than 10%. The inter-assay and intra-assay coefficient of variations determined by quality control samples were ≤15% and ≤10%, respectively. AFC was determined through trans-vaginal ultrasound and defined as the total amount of 2–9 mm follicles in both ovaries. Women with polycystic ovary (PCO) morphology or polycystic ovary syndrome (PCOS) were diagnosed according to the Rotterdam criteria [21]. Other clinical data on age, BMI, and causes of infertility were retrieved from electronic medical records.

### Urine collection and exposure assessment

On the day of ovum pick-up, 10 mL spot urine specimen was collected with a sterile polypropylene container from each participant. The median interval between the day of blood collection for AMH and INHB assays and the day of the urine collection for the phthalate assays were 72 d [interquartile range (IQR): 46–93.5 d]. Urine samples were aliquoted into 2 mL specimens and stored at − 80 °C prior to phthalate metabolites measurement. All urine samples were assayed for eight phthalate metabolites using solid-phase extraction coupled with high-performance liquid chromatography and tandem mass spectrometry, as previously described [22]. The eight metabolites included monomethyl phthalate (MMP), monoethyl phthalate (MEP), mono-n-butyl phthalate (MBP), monobenzyl phthalate (MBzP), MEHP, mono(2-ethyl-5-hydroxyhexyl) phthalate (MEHHP), mono(2-ethyl-5-oxohexyl) phthalate (MEOHP) and mono-n-octyl phthalate (MOP). For each batch of 60–110 samples, one blank, two quality control samples and six standards were processed along with the urine samples. The blank control contained 1-mL water was used to assess the contaminations during sample processing and analysis. Two quality control samples spiked with 5 and 50 ng/mL of the target phthalates, respectively, were used to determine the validation of intraday method accuracy by calculating the recovery. The average recovery for target compounds ranged from 88.06% to 110.93%, and RSD was less than 10.00%. The calibration curve generated from six analytical standards had a linearity of > 0.99, with a range of 0.5–200 ng/mL. If any metabolites in urine samples had much higher concentrations than the linear range of the calibration curves, the samples were re-analyzed after dilution of remaining samples to ensure the accuracy of measurements. The limits of detection (LOD) ranged from 0.01 to 0.04 ng/mL for targeted analytes. Additionally, we calculated the molar sum of the DEHP metabolites (∑DEHP)—MEHP, MEHHP, and MEOHP, by converting the individual metabolite into molar concentration (μmol/L). To standardize the measures, we determined urinary creatinine concentrations using an automated clinical chemistry analyzer.

### Statistical analysis

In descriptive analyses, we used median (IQR) or number (%) to describe the demographic and clinical characteristics of the study population. Urinary phthalate metabolites were standardized by creatinine. The creatinine-adjusted concentrations were expressed as microgram per gram creatinine. We calculated geometric means, medians and selected percentiles to summarize the distributions of phthalate metabolites. Urinary concentrations below the LOD were assigned a value of LOD/√2. Because more than 70% MOP were below the LOD, we dichotomized the concentrations of MOP as either being below or above the LOD and considered it as a dichotomous variable in subsequent analysis.

Multivariable linear regression was performed to estimate the associations of phthalate metabolites with serum AMH and INHB. AMH and INHB levels were natural logarithm transformed to achieve normality. Based on the distribution of the subjects, phthalate metabolites (unadjusted) were categorized into quartiles and estimates for each outcome measure were obtained by comparing each higher quartile to the lowest one (reference category). Tests for trend were performed to explore the potential dose-response relationships between phthalate metabolites and the hormones using the exposure quartiles as ordinal categorized variables with integer values (1–4). The associations between MOP and the hormone measures were also evaluated with concentrations of MOP < LOD as the reference value. We have constructed two types of regression models by adjusting different sets of covariates, and selected covariates based on their biological relevance. Although ethnicity and current smoking were suggested to be potential confounders, they were not considered for inclusion due to their low presentation (< 5%) in the total population. Considering that age and BMI were related to both phthalate exposure and hormone levels as previously outlined [23–25], they were selected to enter the regression models. We considered for urine dilution adjustment by adding creatinine as a separate covariate in the multivariable regression models instead of creatinine-corrected concentrations (i.e. μg/g Cr) because creatinine levels have been suggested to associate with age, gender, race/ethnicity, BMI, muscle mass, diet, activity, etc., thus modeling creatinine-corrected metabolite levels may introduce bias [26, 27]. Therefore, age, BMI and creatinine were entered into model 1 as covariates. Since women diagnosed with PCOS or PCO morphology tend to have altered AMH and INHB levels [28, 29], infertility diagnosis of PCO/PCOS (yes or no) were adjusted in model 2 as a binary variable, together with covariates from model 1. Given that in the regression analysis serum AMH and INHB were ln-transformed, we calculated the percent change with the regression coefficients (β) to allow for easier interpretation of the results. The percent change and corresponding 95% confidence intervals (CI) were calculated as follows: [exp (β)-1]*100, which indicates a percent difference in the outcome comparing each of the higher category of exposure to the lowest one (reference category).

To further explore the potential nonlinearity of the relationship between urinary phthalates and the hormones, we used restricted cubic splines (RCS) with 3 knots at the 5th, 50th, and 95th percentiles of natural logarithm transformed phthalate distributions and set the median level as reference. The optimal number of knots was selected based on model fit and biologic plausibility. We chose the 3-knot RCS function because it had lower Akaike Information Criteria (AIC), which suggests that the model could better explain the observation, while easier for interpretation in the biological context. The location of knots was selected as usually recommended, because it has been suggested to have a little impact on the shape of the dose-response association compared to the number of knots [30]. The Wald chi-square test was used to assess the overall and nonlinear associations between phthalate exposure and the hormones [31]. Taking into account the underlying effect modification by age, we reran the multiple regression models by dividing the participants into young and advanced age groups (< 35 years versus ≥35 years). We examined whether age modifies the effect of urinary phthalate metabolites on serum AMH and INHB levels by adding a product term between metabolite quartiles modeled as ordinal variables and strata of age in the regression models.

Since serum AMH levels could be dichotomized at a cut-off value of 1.1 ng/mL to indicate women with diminished or normal ovarian reserve [32], we additionally explored the effect of urinary phthalate concentrations on this clinically relevant endpoint using multivariable logistic regressions. In addition to hormone biomarkers of ovarian reserve, in the secondary analysis, we fitted multivariable generalized linear models with a Poisson distribution and log-link function to evaluate the associations of urinary phthalate concentrations with AFC. All data analyses were performed using either the Predictive Analytics Suite Workstation (PASW) version 22.0 (IBM Corporation, Armonk, NY) or SAS 9.4 software (SAS Institute, Inc., Cary, NC, USA). Statistical significance was assumed for *P* < 0.05.

## Results

The present study comprised 415 women with an average age of 30 years and BMI of 21.5 kg/m^2^. Most of them were Han (96.9%) and non-smokers (94.9%). A total of 229 women (55.2%) were nulliparous. Nearly half of the subjects underwent IVF or ICSI due to tubal or pelvic infertility (40.0%), followed by male factor (23.9%) and diminished ovarian reserve (13.5%). The median (IQR) concentrations of serum AMH and INHB were 3.90 (1.96–6.99) ng/mL and 82 (59–104) pg/mL, respectively, and AFC was 12 on average. Since eleven women had missing values on serum INHB levels, they were excluded from relevant analysis. Other basic characteristics of the participants were summarized in Table 1.

**Table 1:** Demographics and clinical characteristics of the subjects (*n* = 415)

Characteristics	Data
Age (years)	30 (27–35)
BMI (kg/m^2^)	21.5 (19.8–23.7)
Ethnicity	
Han	402 (96.9)
Other	13 (3.1)
Smoking	
Non-smoker	394 (94.9)
Former smoker	19 (4.6)
Current smoker	2 (0.5)
Gravidity	
Yes	186 (44.8)
No	229 (55.2)
Duration of infertility (years)	3 (1.5–5.0)
IVF/ICSI treatment indication	
Tubal or pelvic factor infertility	166 (40.0)
Ovulation disorders	40 (9.6)
Diminished ovarian reserve	56 (13.5)
Endometriosis	23 (5.5)
Uterine disorders	13 (3.1)
Male factor	99 (23.9)
Unexplained	18 (4.3)
AFC	12 (8–18)
AMH (ng/mL)	3.90 (1.96–6.99)
INHB^a^ (pg/mL)	82 (59–104)

BMI: body mass index; IVF: in vitro fertilization; ICSI: intracytoplasmic sperm injection; AFC: antral follicle count; AMH: anti-Müllerian hormone; INHB: inhibin B

Data are median [IQR (interquartile range)] or number (%)

^a^Serum INHB levels were measured in 404 women

Table 2 presents the creatinine-adjusted concentrations of urinary phthalate metabolites. The detection frequencies of most phthalate metabolites were considerably high, ranging from 95.4% to 100%, except for MOP with 28.7% samples had concentration below the LOD. Metabolite concentrations in the urine varied widely. Among them, MBP had the highest median level (112.74 μg/g Cr), followed by metabolites of DEHP—MEHHP (median 12.30 μg/g Cr), MEHP (median 10.75 μg/g Cr) and MEOHP (median 10.27 μg/g Cr).

**Table 2:** Distribution of urinary phthalate metabolites^a^ (*n* = 415)

Metabolites	% > LOD	GM	Median	Selected percentiles	
				25%	75%
MMP	96.6	7.23	6.86	3.65	15.86
MEP	100	10.73	8.81	4.16	22.28
MBP	100	106.40	112.74	66.61	180.82
MBzP	95.4	0.08	0.07	0.03	0.17
MEHP	98.8	9.99	10.75	5.44	20.68
MEHHP	100	13.40	12.30	8.16	20.46
MEOHP	100	10.42	10.27	6.36	16.91
MOP	28.7	0.04	<LOD	<LOD	0.12
∑DEHP	–	0.13	0.12	0.08	0.20

LOD: the limits of detection; GM: geometric mean; MMP: monomethyl phthalate; MEP: monoethyl phthalate; MBP: monobutyl phthalate; MBzP: monobenzyl phthalate; MEHP: mono(2-ethylhexyl) phthalate; MEHHP: mono(2-ethyl-5-hydroxyhexyl) phthalate; MEOHP: mono(2-ethyl-5-oxohexyl) phthalate; MOP: mono-n-octyl phthalate; DEHP: di(2-ethylhexyl) phthalate

∑DEHP: Molar sum of DEHP metabolites (MEHP, MEHHP and MEOHP) expressed in μmol/g creatinine (Cr)

% > LOD: Phthalate metabolites above the limits of detection

^a^Urinary phthalate metabolites concentrations were creatinine adjusted (μg/g Cr)

Overall, no significant dose-response associations were observed between urinary phthalate metabolites and serum AMH in linear models, after adjusting for age, BMI and creatinine (Table 3). Similar results were obtained when additionally adjusted for PCO/PCOS diagnosis (yes or no) in model 2, except for an increase in AMH levels comparing MOP > LOD to those below the LOD. Moreover, we examined the validity of the linearity assumption between phthalates and serum AMH with restricted cubic spline analysis. In spline regression models, the overall and nonlinear spline terms were both non-significant, thus further corroborating our null findings between urinary phthalates and serum AMH (Additional file 1: Figure S1). In the age-stratified analysis, the associations between phthalates and AMH were not modified by age (*P* for interaction > 0.05). Urinary concentrations of metabolites were consistently not related to serum AMH neither among women < 35 years nor among those ≥35 years (Additional file 2: Table S1).

**Table 3:** Associations between urinary phthalate metabolite concentrations and serum AMH^1^ in multivariable linear models (*n* = 415)

Metabolite	Model 1^4^	Model 2^5^
	β (95% CI)	β (95% CI)
MMP^2^		
1^6^ (< 5.18)	Ref	Ref
2 (5.18–12.21)	− 0.06 (− 0.29, 0.17)	0.03 (− 0.19, 0.24)
3 (12.21–25.78)	− 0.10 (− 0.34, 0.14)	0.05 (− 0.17, 0.27)
4 (> 25.78)	0.02 (− 0.22, 0.25)	0.04 (− 0.18, 0.26)
MEP^2^		
1^6^ (< 6.02)	Ref	Ref
2 (6.02–12.80)	− 0.10 (− 0.35, 0.14)	− 0.08 (− 0.30, 0.15)
3 (12.80–33.98)	− 0.05 (− 0.30, 0.20)	− 0.10 (− 0.32, 0.13)
4 (> 33.98)	− 0.08 (− 0.33, 0.17)	− 0.12 (− 0.34, 0.11)
MBP^2^		
1^6^ (< 73.85)	Ref	Ref
2 (73.85–184.55)	**0.28 (0.04, 0.52)**	0.18 (−0.03, 0.40)
3 (184.55–342.12)	0.01 (− 0.24, 0.27)	− 0.08 (− 0.31, 0.16)
4 (> 342.12)	0.18 (− 0.10, 0.47)	0.11 (− 0.15, 0.37)
MBzP^2^		
1^6^ (< 0.035)	Ref	Ref
2 (0.035–0.102)	0.04 (−0.20, 0.27)	0.04 (−0.17, 0.26)
3 (0.102–0.27)	−0.06 (− 0.31, 0.20)	0.02 (− 0.21, 0.25)
4 (> 0.27)	0.12 (−0.14, 0.38)	0.15 (−0.08, 0.38)
MEHP^2^		
1^6^ (< 6.95)	Ref	Ref
2 (6.95–17.21)	0.12 (−0.11, 0.35)	0.10 (−0.11, 0.31)
3 (17.21–36.01)	0.06 (− 0.18, 0.31)	0.01 (−0.21, 0.24)
4 (> 36.01)	0.20 (−0.05, 0.45)	0.16 (−0.07, 0.39)
MEHHP^2^		
1^6^ (< 10.94)	Ref	Ref
2 (10.94–19.09)	0.08 (−0.16, 0.33)	0.09 (−0.14, 0.31)
3 (19.09–34.68)	0.12 (−0.15, 0.39)	0.12 (−0.13, 0.36)
4 (> 34.68)	0.24 (−0.04, 0.52)	0.17 (−0.08, 0.43)
MEOHP^2^		
1^6^ (< 7.41)	Ref	Ref
2 (7.41–15.34)	0.02 (−0.22, 0.26)	−0.03 (− 0.26, 0.19)
3 (15.34–27.72)	−0.03 (− 0.29, 0.22)	− 0.05 (− 0.29, 0.18)
4 (> 27.72)	0.16 (− 0.11, 0.44)	0.05 (− 0.21, 0.30)
∑DEHP^2^		
1^6^ (< 0.10)	Ref	Ref
2 (0.10–0.19)	0.14 (−0.10, 0.39)	0.07 (−0.15, 0.29)
3 (0.19–0.35)	0.01 (−0.25, 0.26)	−0.02 (− 0.25, 0.21)
4 (> 0.35)	0.19 (−0.08, 0.46)	0.12 (− 0.13, 0.36)
MOP^3^	0.16 (−0.02, 0.34)	**0.20 (0.04, 0.37)**

Statistically significant results comparing a specific category to the reference are bolded

^1^Serum AMH levels were natural logarithm transformed

^2^Phthalate metabolite concentrations were categorized into quartiles

^3^Dichotomous variable based on above/below limits of detection

^4^Model 1 was adjusted for age, BMI and creatinine

^5^Model 2 was adjusted for age, BMI, creatinine and PCO/PCOS diagnosis (yes or no)

^6^Reference category

In the linear regression models adjusted for age, BMI and creatinine, we found a suggestive trend for lower serum INHB among women with higher urinary MEHP concentrations (*P-*trend = 0.06) (Table 4). Compared to the lowest quartile, women in the highest quartile of MEHP had a significant decrease of − 17.55% (95% CI: − 31.34%, − 0.90%) in INHB levels. When controlling for the covariates in model 2, the dose-response trend between MEHP and serum INHB reached significance (*P-*trend = 0.04), with a larger decrease of percent difference in quartile 4 from quartile 1 (− 18.29%; 95% CI: − 31.89%, − 1.98%). For MEOHP, significant decrease in serum INHB was observed across the second to fourth quartiles compared with quartile one in adjusted model 2. The adjusted differences in INHB levels for quartile 2, 3 and 4 compared with quartile 1 of MEOHP were − 23.74% (95% CI: −35.85%, − 9.24%), − 19.91% (95% CI: −33.30%, − 3.82%) and − 20.23% (95% CI: −34.43%, − 2.96%), respectively. The largest decrease found in quartile two suggested that there might exist a threshold value. Decrease in INHB seemed to reach a plateau when concentrations exceeded the threshold, reflecting a potential nonlinear relationship. Among the remaining six phthalate metabolites and ∑DEHP, no consistent change in serum INHB levels was observed. In the age-stratified analyses, women in the younger subgroup had apparent decrease in INHB levels for MEHP, MEOHP and ∑DEHP quartiles, whereas women ≥35 years had no significant change in INHB levels (Additional file 3: Table S2). Although the product terms were not statistically significant (*P* for interaction > 0.05), this finding may imply a potential effect modification by age.

**Table 4:** Associations between urinary phthalate metabolite concentrations and serum INHB^1^ in multivariable linear models (*n* = 404)

Metabolite	Model 1^4^	Model 2^5^
	Percent change^6^ (95% CI)	Percent change^6^ (95% CI)
MMP^2^		
1^7^ (< 5.18)	Ref	Ref
2 (5.18–12.21)	−2.47 (− 17.72, 15.49)	− 0.30 (− 15.80, 17.94)
3 (12.21–25.78)	−1.00 (− 16.72, 17.70)	3.15 (− 13.24, 22.75)
4 (> 25.78)	4.71 (− 11.75, 24.23)	5.23 (− 11.13, 24.73)
MEP^2^		
1^7^ (< 6.02)	Ref	Ref
2 (6.02–12.80)	−4.40 (− 19.83, 14.00)	− 3.92 (− 19.27, 14.34)
3 (12.80–33.98)	3.36 (− 13.50, 23.49)	1.82 (− 14.62, 21.41)
4 (> 33.98)	0.50 (− 16.31, 20.56)	− 0.80 (− 17.22, 18.89)
MBP^2^		
1^7^ (< 73.85)	Ref	Ref
2 (73.85–184.55)	− 9.79 (− 24.19, 7.47)	−11.84 (− 25.84, 4.81)
3 (184.55–342.12)	− 16.56 (− 30.65, 0.50)	**−18.62 (− 32.23, − 2.18)**
4 (> 342.12)	− 14.02 (− 30.16, 5.87)	−15.46 (− 31.20, 3.87)
MBzP^2^		
1^7^ (< 0.035)	Ref	Ref
2 (0.035–0.102)	−9.43 (− 23.59, 7.36)	− 9.34 (− 23.43, 7.25)
3 (0.102–0.27)	− 7.78 (− 23.28, 10.96)	−5.45 (− 21.34, 13.54)
4 (> 0.27)	3.46 (− 13.84, 24.23)	4.39 (− 12.89, 25.23)
MEHP^2^		
1^7^ (< 6.95)	Ref	Ref
2 (6.95–17.21)	− 13.32 (− 26.80, 2.53)	− 13.58 (− 26.88, 2.12)
3 (17.21–36.01)	− 12.89 (− 26.95, 3.87)	−14.02 (− 27.75, 2.33)
4 (> 36.01)	**− 17.55 (− 31.34, − 0.90)**	− 18.29 (− 31.89, − 1.98)*
MEHHP^2^		
1^7^ (< 10.94)	Ref	Ref
2 (10.94–19.09)	−7.78 (− 23.05, 10.52)	− 7.41 (− 22.59, 10.74)
3 (19.09–34.68)	1.61 (−16.56, 23.61)	1.71 (− 16.31, 23.61)
4 (> 34.68)	− 3.63 (− 21.42, 18.18)	−5.07 (− 22.43, 16.18)
MEOHP^2^		
1^7^ (< 7.41)	Ref	Ref
2 (7.41–15.34)	**− 22.74 (− 35.14, − 7.87)**	**−23.74 (− 35.85, − 9.24)**
3 (15.34–27.72)	**− 19.59 (− 33.17, − 3.25)**	**−19.91 (− 33.30, − 3.82)**
4 (> 27.72)	−17.80 (− 32.56, 0.20)	**− 20.23 (− 34.43, − 2.96)**
∑DEHP^2^		
1^7^ (< 0.10)	Ref	Ref
2 (0.10–0.19)	−9.43 (− 23.97, 7.90)	−11.04 (− 25.25, 5.87)
3 (0.19–0.35)	−13.58 (− 28.18, 3.87)	− 14.10 (− 28.39, 3.15)
4 (> 0.35)	−12.10 (− 27.75, 6.82)	− 13.76 (− 28.97, 4.71)
MOP^3^	−2.76 (− 14.62, 10.74)	−1.78 (− 13.67, 11.74)

*Tests for linear trend with *P*-value < 0.05. Statistically significant results comparing a specific category to the reference are bolded

^1^Serum INHB levels were natural logarithm transformed

^2^Phthalate metabolite concentrations were categorized into quartiles

^3^Dichotomous variable based on above/below limits of detection

^4^Model 1 was adjusted for age, BMI and creatinine

^5^Model 2 was adjusted for age, BMI, creatinine and PCO/PCOS diagnosis (yes or no)

^6^Percent change and 95% CI were calculated as follows: [exp (β)-1]*100

^7^Reference category

**Fig. 1:**
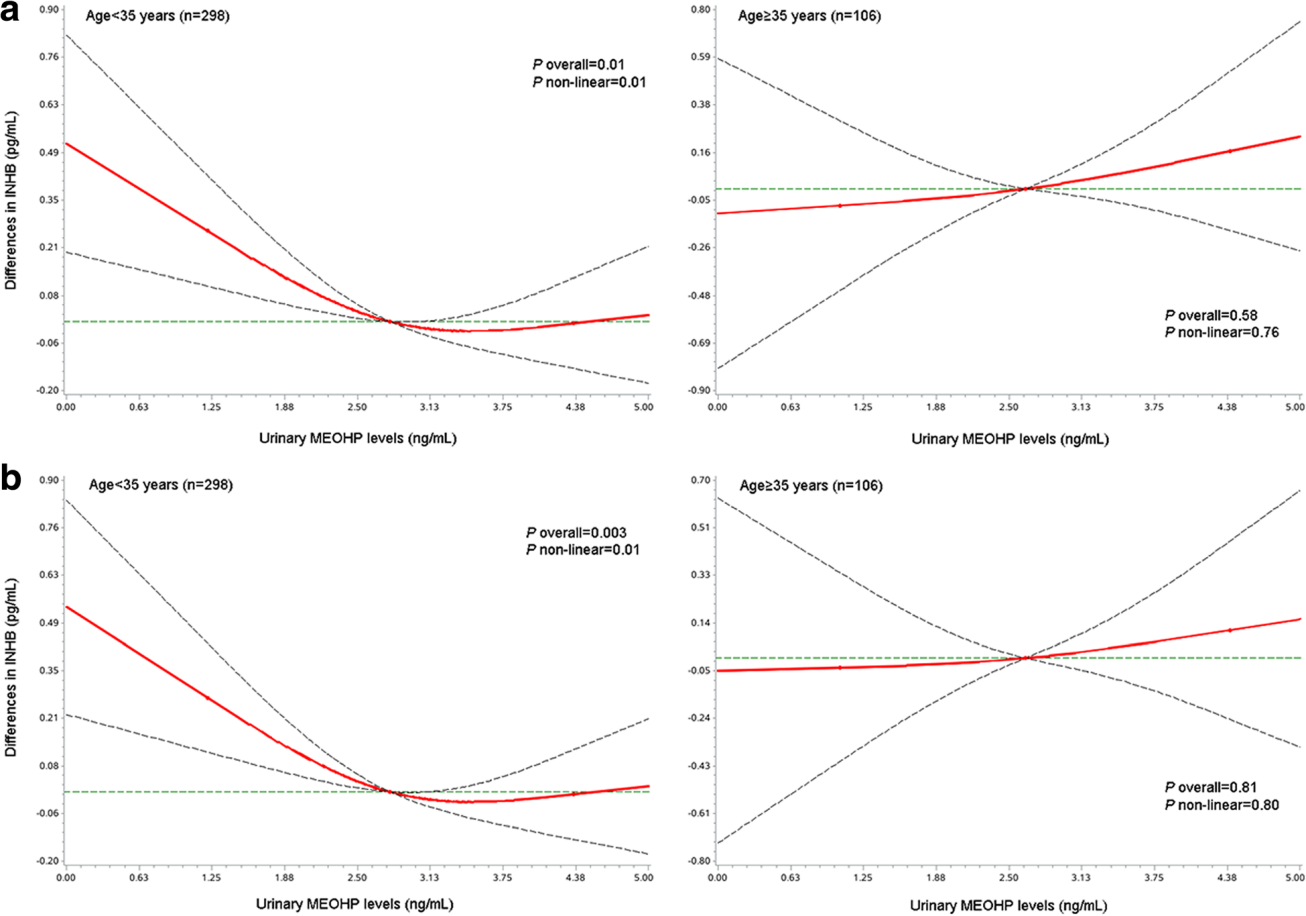
Adjusted differences (red line) in serum INHB levels by urinary concentrations of phthalate metabolites. Age-stratified (< 35 versus ≥35 years) multivariable linear regression models for both ln-transformed phthalates and INHB levels were adjusted for age, BMI, and creatinine (**a**), and additionally adjusted for PCO/PCOS diagnosis (**b**). Urinary phthalate metabolites were modeled as restricted cubic splines with knots placed at the 5th, 50th, and 95th percentiles, and the reference level (green line) was set at the median. Dashed lines = 95% CI; dots = knots

When the metabolites were modeled as continuous variables in the spline analysis, we identified a nonlinear relationship between MEOHP exposure and serum INHB among women < 35 years of age (Fig. 1). The test for the overall association between MEOHP and INHB in the younger subgroup was significant (*P* for overall association < 0.05), which means whatever the shape of the association is, MEOHP was significantly correlated with INHB. The null hypothesis of the test for nonlinearity that assumed a linear relationship between MEOHP and INHB was rejected (*P* for nonlinear association = 0.01), suggesting there exists a nonlinear association. Collectively, we observed that within a range of relatively low levels of exposure, serum INHB was negatively associated with urinary MEOHP in a dose dependent pattern. However, when a threshold level was met, the decline in INHB was attenuated.

In logistic regression models comparing women with low (defined as an AMH value of < 1.1 ng/mL) versus normal ovarian reserve, urinary phthalate concentrations were not associated with an increased odds of DOR after adjusting for potential confounders (Additional file 4: Table S3). In the secondary analysis using AFC as a biomarker of ovarian reserve, a positive dose-response relationship between quartiles of urinary MEHHP and MEOHP levels in relation to AFC was observed with the adjustment for age, BMI and creatinine (*P*-trend for MEHHP = 0.001; *P*-trend for MEOHP = 0.004). However, when further adjusting for diagnosis of PCO/PCOS in model 2, these positive associations appeared non-significant (Additional file 5: Table S4).

## Discussion

Although a growing number of in vitro and animal studies are emerging linking phthalates with altered ovarian reserve, we found largely null associations between urinary phthalate metabolites and serum AMH levels, considered one of the best markers of ovarian reserve, in this large retrospective study comprised of 415 women undergoing IVF. Another major finding in this study was that urinary MEHP was associated with lower serum INHB levels. We also found evidence of a nonlinear relationship between MEOHP concentrations and INHB.

As the number of activated follicles is dependent upon primordial follicle pool, serum AMH is considered to reflect the ovarian reserve, despite the fact that AMH is produced by small growing follicles rather than by non-growing primordial follicles [33]. Therefore, though AMH is a relatively acute marker of ovarian reserve currently available in clinical practice, its indirect reflection of primordial follicles in nature may contribute to the lack of association between serum AMH and phthalate exposure observed in our study. In one study comprising menopausal women, urinary concentrations of MEHHP and MEOHP were related to earlier age at natural menopause [34]. The ovarian reserve declines with increasing age, culminating in menopause with a virtual exhaustion of follicle numbers. Therefore, to reveal the actual effect of phthalate exposure on ovarian aging, a more direct marker or the endpoint of ovarian pool exhaustion, for example menopause, might be needed. Another explanation for our null findings is that the accelerated follicle loss caused by phthalates may date back to early life exposure [1]. Given that fetal organ development during pregnancy is susceptible to the intrauterine environment, it is possible that prenatal exposure to phthalates may heighten the risk of reduced ovarian reserve in adulthood. In support of our speculation, one animal study demonstrated that maternal exposure to DEHP has sped up the recruitment of primordial follicles in F1 and F2 offspring, leading to premature ovarian failure [35]. Hart et al. reported that girls who experienced in utero exposure to MEP exhibited lower serum AMH levels in their adolescence, suggesting that early life exposure has the potential to modify the trajectory of ovarian reserve in adult life [36]. Thus, it is possible that with a prospective longitudinal study design and exposure assessment at early life stages, the effect of phthalates on ovarian aging would be elucidated. Finally, we observed a positive association between urinary MOP and higher levels of AMH, which was the sole finding that reached statistical significance. Nevertheless, it should be noted that because of the low levels and detection rates of urinary MOP, as well as the multiple comparisons made in the analysis, the result might be spurious or a chance finding.

As a functional unit of the ovary, the abnormal growth and atresia of antral follices may cause defects in hormone production. Evidence from toxicological studies has established antral follicles as a target of phthalates. With an in vitro culture system, Gupta et al. demonstrated that DEHP and its bioactive metabolite MEHP had the potential to inhibit antral follicle growth and disrupt steroidogenesis manifested as a decline in estradiol production [37]. Moreover, DEHP were also shown to induce follicle atresia in cultured mouse antral follicles via mechanisms involving imbalanced pro- and anti-apoptotic pathways and dysregulated expression of genes involved in cell cycle [11]. Consequently, our observations of decreased serum INHB in relation to MEHP might be a result of increased astresia and growth arrest of antral follicles. A paucity of human data has been gathered regarding the effect of phthalate exposure on ovarian reserve. One prospective cohort study involving women seeking infertility treatment has demonstrated that a significant decrease in AFC was associated with higher urinary concentrations of DEHP metabolites [16]. Our findings of reduced INHB levels and higher MEHP is corroborated by their findings to some extent, because AFC determined by trans-vaginal ultrasound are comprised of follicles with a size of 2 to 10 mm, which is equivalent to the follicles secreting INHB [38]. It has been established that women with PCOS has higher concentrations of serum INHB which reflects the increased number of antral follicles [29]. Thus, it is plausible that the associations between MEHP and INHB became more evident when we additionally controlled for PCO/PCOS diagnosis. In addition, we identified a nonlinear association between MEOHP and serum INHB, which appeared more apparent among younger women. Within a low level of exposure, urinary MEOHP was inversely correlated with INHB until it reached a potential threshold value at the second quartile. This finding is also supported by a research showing a similar decrease in AFC after a threshold level of DEHP metabolites was met [16]. Nonlinear effect is not uncommon in studies of natural hormones and EDCs. Grande et al. revealed a threshold effect of DEHP exposure during gestation and lactation on the delay of pubertal onset in female offspring [39]. Similarly, in adult mice, DEHP exposure induced prolonged estrous cycles in a non-monotonic dose response [40]. Although the specific mechanisms by which phthalates affect ovarian function remains unclear, there is evidence that phthalates could exert their effects through binding steroid receptors [41]. Thus, one possible explanation for the attenuated decrease in INHB at higher MEOHP level might be that the receptors at the target tissue or cells are gradually saturated, leading to the observed nonlinear biological effect. In addition, our findings do show some cause for concern over the low-dose effect of phthalate exposure and need further verification in both animal and human studies. In line with the study reported by Messerlian and Souter et al. [16], younger women were at higher risk of phthalate-induced ovarian toxicity, as indicated by the stratified analysis. Among women of advanced age, the null associations between phthalate exposure and INHB may be explained by the fact that the effect magnitude of phthalate is relatively small when compared with age which is of greater value in influencing ovarian function. In contrast, younger women may be more sensitive to other factors, and the influence of age has not preponderated over others. Thus, the effect of phthalates appeared evident.

The discrepancy between serum AMH and INHB in relation to phthalate metabolites was in agreement with an animal study exploring the effect of acute DEHP exposure on fecundability and ovarian aging [40]. In this study, adult mice treated with DEHP orally for 10 days exhibited dysregulated folliculogenesis and decreased serum levels of INHB at 9-months postdosing, while serum AMH levels were not affected. The differences in hormone results were explained by the observation that acute exposure to DEHP did not alter the number of preantral follicles, but increased the percentage of atretic antral follicles. Still, the divergent finding was somewhat puzzling. Nevertheless, several potential explanations were considered. Although both serum AMH and INHB are secreted by small growing follicles, follicles that contribute the most to AMH and INHB levels are differed by diameter. Follicles of 1–2 mm in diameter are probably the main contributors to serum AMH levels with the evidence that the intrafollicular concentrations of AMH decreased gradually with increasing follicle diameters [18], concomitant with the observation that granulosa cells of secondary, preantral and small antral follicles < 4 mm in diameter expressed the highest level of AMH [42]. In contrast, concentrations of INHB increased with the growth of follicles until they reached a diameter of 9 mm [43]. Hence, larger antral follicles at later developmental stage constitute the primary source of serum INHB. Based on these facts, if a patient whose AFC is mostly represented by small follicles (i.e. 1–2 mm), while phthalates target larger antral follicles, the discrepant findings between serum AMH and INHB relative to urinary phthalate concentrations might occur. Another explanation is probably that women included in the present study had different status of ovarian reserve manifested as altered concentrations of gonadotropin hormones [i.e. FSH and luteal hormone (LH)] in the early follicular phase. While AMH function as a paracrine factor independent of endogenous hormones, the biosynthesis of INHB is regulated by FSH and LH, and involved in the pituitary gland feedback loop [44]. Therefore, the different characteristics of ovarian reserve in the study population may be a potential reason for inconsistency between the AMH and INHB results.

In the secondary analysis, the findings of AFC and phthalates were inconsistent with that of INHB which is contrary to our expectation. The divergent observations between AFC and INHB might be partially ascribed to their different characteristics in reflecting ovarian reserve. Because AFC could not distinguish healthy from atretic follicles [18], when phthalate exposure increases follicle atresia and consequently affects hormone production, the occurrence of the discrepant results seems plausible.

Our relatively large sample size of 415 women gave us more than 85% power to detect a correlation between phthalate concentrations and hormone levels when the correlation coefficient was assumed 0.15 and the two-sided significance level was set at 0.05. With regard to the serum AMH and INHB measurement in the present study, the well-established automated assay platform in routine clinical practice minimized the inter- and intra-operator variability. Meanwhile, it is worth noting that our retrospective study design precluded us from drawing a causality of exposure and altered ovarian function. Additionally, since urine collection for exposure measurement was conducted up to 4 months after AMH and INHB assays, there is a possibility of reverse causation. Phthalates could be found in a wide range of personal care products, foodstuffs, certain types of oral medications and medical devices [4], thus we could not exclude the possibility that urinary phthalate concentrations might be influenced by changes in daily care routines and dietary habits, as well as medical interventions during the 4-month period. Additionally, misclassification of exposure was likely to occur not only from timing of exposure and outcome but also from our single measurement of spot urine because of short half-life of phthalates and variability in individual behaviors. However, it has been suggested that one spot urine measurement has moderate capacity to reflect average exposure within 4 months (sensitivity ranging from 0.58 to 0.77), when a surrogate category analysis was performed [45]. Given that the median time intervals between blood collection for AMH and INHB assays and urine collection for phthalate measurement were 72 d (IQR: 46–93.5 d) in the study, the single measurement might allow for a moderately reliable ranking of a few months exposure preceding the urine collection. Finally, our findings drawn from women undergoing IVF may not extrapolate to the general population, and the possibility of bias from uncontrolled confounding may also exist.

## Conclusions

In this large retrospective study, we provided evidence for a negative association between higher urinary concentrations of certain phthalate metabolites and serum INHB levels, suggesting an adverse effect of phthalates exposure on growing antral follicles. However, little evidence of an association was observed between urinary phthalates and serum AMH. Whether phthalates exposure at environmentally level will pose a risk for ovarian reserve could not be determined from the present data, and further studies with a prospective design and more direct indicators of primordial follicle pool might reveal the potential effect.

## Additional files

Additional file 1: Figure S1. Adjusted differences (red line) in serum AMH levels by urinary concentrations of phthalate metabolites. Multivariable linear regression models for both ln-transformed phthalates and AMH levels were adjusted for age, BMI, and creatinine. Urinary phthalate metabolites were modeled as restricted cubic splines with knots placed at the 5th, 50th, and 95th percentiles, and the reference level (green line) was set at the median. Dashed lines = 95% CI; dots = knots. (TIFF 247 kb)

Additional file 2: Table S1. Associations between urinary phthalate metabolites and serum AMH^1^ in multivariable linear models stratified by age. (DOCX 17 kb)

Additional file 3: Table S2. Associations between urinary phthalate metabolites and serum INHB^1^ in multivariable linear models stratified by age. (DOCX 18 kb)

Additional file 4: Table S3. Adjusted odds ratios (95% CI) for polycystic ovarian morphology (PCOM) and diminished ovarian reserve (DOR) by urinary phthalate metabolites (*n* = 415). (DOCX 15 kb)

Additional file 5: Table S4. Associations between urinary phthalate metabolites and AFC in multivariable generalized linear models (*n* = 415). (DOCX 14 kb)

## Abbreviations

∑DEHP: Molar sum of DEHP metabolites (MEHP, MEHHP and MEOHP) expressed in μmol/L
AFC: Antral follicle count
AIC: Akaike information criteria
AMH: Anti-Müllerian hormone
BMI: Body mass index
DBP: Di-n-butyl phthalate
DEHP: Di(2-ethylhexyl) phthalate
E_2_: Estradiol
EDCs: Endocrine-disrupting chemicals
ELISA: Enzyme-linked immunuosorbent assay
FSH: Follicle-stimulating hormone
ICSI: Intracytoplasmic sperm injection
INHB: Inhibin B
IQR: Interquartile range
IVF: in vitro fertilization
LH: Luteal hormone
LOD: The limits of detection
MBP: Monobutyl phthalate
MBzP: Monobenzyl phthalate
MEP: Monoethyl phthalate
MEHP: Mono(2-ethylhexyl) phthalate
MEHHP: Mono(2-ethyl-5-hydroxyhexyl) phthalate
MEOHP: Mono(2-ethyl-5-oxohexyl) phthalate
MMP: Monomethyl phthalate
MOP: Mono-n-octyl phthalate
PCO: Polycystic ovary
PCOS: Polycystic ovary syndrome
RCS: Restricted cubic spline
