# Supplementary figures and images for: Urinary phthalate metabolites in relation to serum anti-Müllerian hormone and inhibin B levels among women from a fertility center: a retrospective analysis
Source: Reprod Health. 2018 Feb 23;15:33. doi: 10.1186/s12978-018-0469-8 (PMC5824533; doi:10.1186/s12978-018-0469-8)

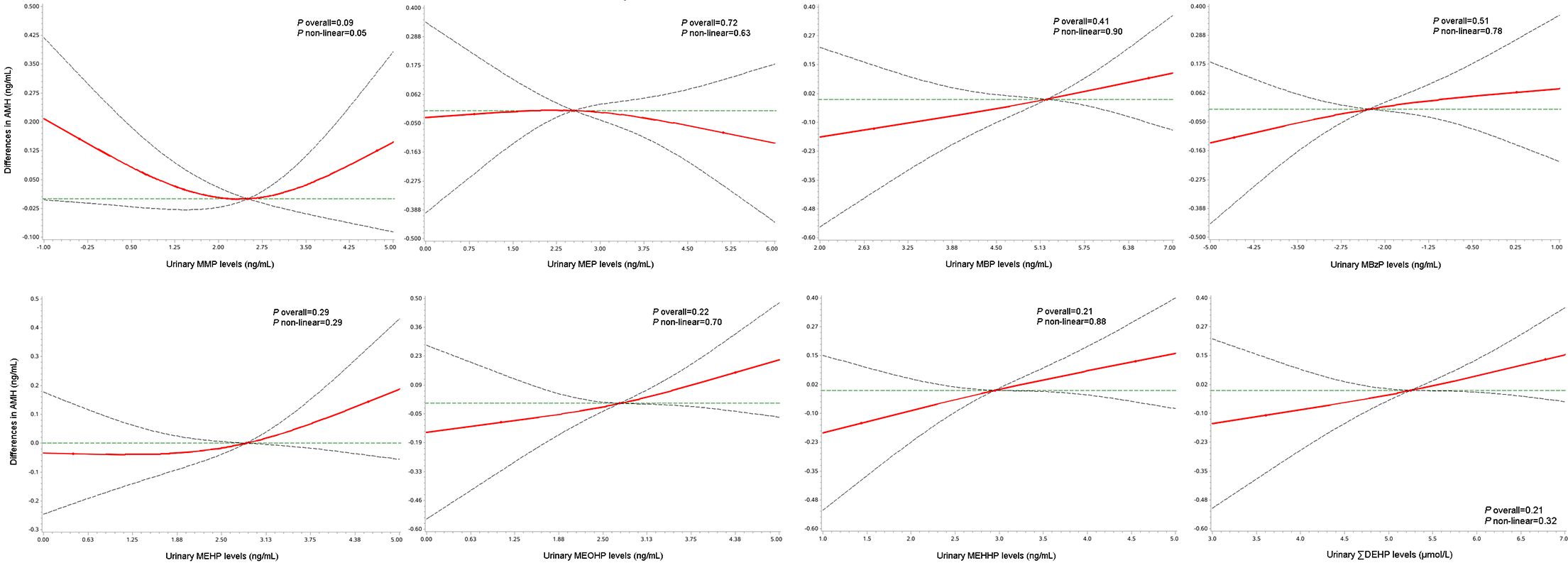

Supplement: Supplementary file 1 — Adjusted differences (red line) in serum AMH levels by urinary concentrations of phthalate metabolites. Multivariable linear regression models for both ln-transformed phthalates and AMH levels were adjusted for age, BMI, and creatinine. Urinary phthalate metabolites were modeled as restricted cubic splines with knots placed at the 5th, 50th, and 95th percentiles, and the reference level (green line) was set at the median. Dashed lines = 95% CI; dots = knots. (TIFF 247 kb) [file 12978_2018_469_MOESM1_ESM.tif]
