# Supplementary material for: Urinary phthalate metabolites in relation to serum anti-Müllerian hormone and inhibin B levels among women from a fertility center: a retrospective analysis
Source: Reprod Health. 2018 Feb 23;15:33. doi: 10.1186/s12978-018-0469-8 (PMC5824533; doi:10.1186/s12978-018-0469-8)
Supplement: Supplementary file 2 — Associations between urinary phthalate metabolites and serum AMH1 in multivariable linear models stratified by age. (DOCX 17 kb) [file 12978_2018_469_MOESM2_ESM.docx]

| **Table S1 Associations between urinary phthalate metabolites and serum AMH^1^ in multivariable linear models stratified by age.** | | | |
| --- | --- | --- | --- |
| Metabolite | <35 years (n=306) |  | ≥35 years (n=109) |
|  | β (95% CI) |  | β (95% CI) |
| MMP^2^ |  |  |  |
| 1^4^ (<5.18) | Ref |  | Ref |
| 2 (5.18-12.21) | −0.002 (−0.28, 0.28) |  | −0.17 (−0.63, 0.28) |
| 3 (12.21-25.78) | −0.08 (−0.35, 0.19) |  | −0.11 (−0.66, 0.44) |
| 4 (>25.78) | 0.04 (−0.23, 0.32) |  | −0.02 (−0.50, 0.47) |
| MEP^2^ |  |  |  |
| 1^4^ (<6.02) | Ref |  | Ref |
| 2 (6.02-12.80) | −0.20 (−0.48, 0.09) |  | 0.09 (−0.43, 0.61) |
| 3 (12.80-33.98) | −0.18 (−0.47, 0.12) |  | 0.24 (−0.23, 0.72) |
| 4 (>33.98) | −0.14 (−0.43, 0.16) |  | −0.01 (−0.51, 0.50) |
| MBP^2^ |  |  |  |
| 1^4^ (<73.85) | Ref |  | Ref |
| 2 (73.85-184.55) | **0.29 (0.02, 0.57)** |  | 0.19 (−0.29, 0.68) |
| 3 (184.55-342.12) | −0.12 (−0.41, 0.17) |  | 0.38 (−0.16, 0.92) |
| 4 (>342.12) | 0.08 (−0.25, 0.41) |  | 0.36 (−0.22, 0.94) |
| MBzP^2^ |  |  |  |
| 1^4^ (<0.035) | Ref |  | Ref |
| 2 (0.035-0.102) | −0.16 (−0.43, 0.12) |  | **0.59 (0.13, 1.06)** |
| 3 (0.102-0.27) | −0.11 (−0.40, 0.19) |  | 0.06 (−0.45, 0.57) |
| 4 (>0.27) | −0.01 (−0.29, 0.28) |  | 0.46 (−0.08, 1.01) |
| MEHP^2^ |  |  |  |
| 1^4^ (<6.95) | Ref |  | Ref |
| 2 (6.95-17.21) | 0.07 (−0.20, 0.34) |  | 0.22 (−0.26, 0.70) |
| 3 (17.21-36.01) | −0.03 (−0.30, 0.25) |  | 0.30 (−0.24, 0.83) |
| 4 (>36.01) | 0.13 (−0.17, 0.42) |  | 0.37 (−0.13, 0.87) |
| MEHHP^2^ |  |  |  |
| 1^4^ (<10.94) | Ref |  | Ref |
| 2 (10.94-19.09) | 0.03 (−0.25, 0.32) |  | 0.15 (−0.35, 0.64) |
| 3 (19.09-34.68) | 0.03 (−0.28, 0.34) |  | 0.44 (−0.14, 1.03) |
| 4 (>34.68) | 0.20 (−0.13, 0.52) |  | 0.41 (−0.15, 0.96) |
| MEOHP^2^ |  |  |  |
| 1^4^ (<7.41) | Ref |  | Ref |
| 2 (7.41-15.34) | −0.07 (−0.35, 0.22) |  | 0.24 (−0.25, 0.73) |
| 3 (15.34-27.72) | −0.12 (−0.41, 0.18) |  | 0.26 (−0.27, 0.79) |
| 4 (>27.72) | 0.10 (−0.22, 0.41) |  | 0.40 (−0.17, 0.97) |
| ∑DEHP^2^ |  |  |  |
| 1^4^ (<0.10) | Ref |  | Ref |
| 2 (0.10-0.19) | 0.09 (−0.19, 0.37) |  | 0.28 (−0.20, 0.77) |
| 3 (0.19-0.35) | −0.10 (−0.39, 0.19) |  | 0.30 (−0.22, 0.83) |
| 4 (>0.35) | 0.15 (−0.16, 0.46) |  | 0.33 (−0.21, 0.88) |
| MOP^3^ | 0.11 (−0.10, 0.32) |  | 0.35 (−0.02, 0.73) |
| Statistically significant results comparing a specific category to the reference are bolded. | | | |
| Models were adjusted for age, BMI and creatinine. | | | |
| ^1^Serum AMH levels were natural logarithm transformed. | | | |
| ^2^Phthalate metabolite concentrations were categorized into quartiles. | | | |
| ^3^Dichotomous variable based on above/below limits of detection. | | | |
| ^4^Reference category. | | | |
