# Supplementary material for: Urinary phthalate metabolites in relation to serum anti-Müllerian hormone and inhibin B levels among women from a fertility center: a retrospective analysis
Source: Reprod Health. 2018 Feb 23;15:33. doi: 10.1186/s12978-018-0469-8 (PMC5824533; doi:10.1186/s12978-018-0469-8)
Supplement: Supplementary file 3 — Associations between urinary phthalate metabolites and serum INHB1 in multivariable linear models stratified by age. (DOCX 18 kb) [file 12978_2018_469_MOESM3_ESM.docx]

| **Table S2 Associations between urinary phthalate metabolites and serum INHB^1^ in multivariable linear models stratified by age.** | | | |
| --- | --- | --- | --- |
| Metabolite | <35 years (n=298) |  | ≥35 years (n=106) |
|  | Percent change^4^ (95% CI) |  | Percent change^4^ (95% CI) |
| MMP^2^ |  |  |  |
| 1^5^ (<5.18) | Ref |  | Ref |
| 2 (5.18-12.21) | -3.44 (-19.83, 16.30) |  | -3.15 (-33.63, 41.48) |
| 3 (12.21-25.78) | 4.81 (-12.54, 25.48) |  | -28.54 (-54.66, 12.64) |
| 4 (>25.78) | -2.66 (-18.86, 16.88) |  | 25.11 (-16.39, 87.20) |
| MEP^2^ |  |  |  |
| 1^5^ (<6.02) | Ref |  | Ref |
| 2 (6.02-12.80) | -7.60 (-23.36, 11.52) |  | 2.12 (-34.69, 59.84) |
| 3 (12.80-33.98) | -1.19 (-18.78, 20.20) |  | 9.75 (-26.73, 64.38) |
| 4 (>33.98) | -4.97 (-21.96, 15.84) |  | 8.76 (-29.95, 69.05) |
| MBP^2^ |  |  |  |
| 1^5^ (<73.85) | Ref |  | Ref |
| 2 (73.85-184.55) | -12.37 (-27.24, 5.44) |  | -4.50 (-37.12, 45.21) |
| 3 (184.55-342.12) | **-24.80 (-38.12, -8.70)** |  | 17.00 (-26.43, 86.08) |
| 4 (>342.12) | -19.27 (-35.34, 0.80) |  | 0.80 (-38.92, 66.36) |
| MBzP^2^ |  |  |  |
| 1^5^ (<0.035) | Ref |  | Ref |
| 2 (0.035-0.102) | **-21.49 (-34.36, -6.11)** |  | 38.26 (-7.78, 107.09) |
| 3 (0.102-0.27) | -8.33 (-24.57, 11.40) |  | -3.44 (-34.10, 49.33) |
| 4 (>0.27) | -8.33 (-24.12, 10.85) |  | 48.29 (-7.32, 137.03) |
| MEHP^2^ |  |  |  |
| 1^5^ (<6.95) | Ref |  | Ref |
| 2 (6.95-17.21) | -14.79 (-28.82, 2.02) |  | -7.87 (-38.86, 38.82) |
| 3 (17.21-36.01) | -14.02 (-28.39, 3.25) |  | -9.15 (-42.36, 43.19) |
| 4 (>36.01) | **-21.73 (-35.66, -4.78)*** |  | -4.30 (-38.49, 48.88) |
| MEHHP^2^ |  |  |  |
| 1^5^ (<10.94) | Ref |  | Ref |
| 2 (10.94-19.09) | -13.41 (-28.75, 5.13) |  | 1.71 (-34.30, 57.46) |
| 3 (19.09-34.68) | -6.01 (-23.81, 15.95) |  | 18.06 (-28.25, 94.45) |
| 4 (>34.68) | -12.63 (-29.88, 8.87) |  | 20.80 (-25.32, 95.42) |
| MEOHP^2^ |  |  |  |
| 1^5^ (<7.41) | Ref |  | Ref |
| 2 (7.41-15.34) | **-30.86 (-42.48, -17.72)** |  | 2.94 (-32.97, 58.09) |
| 3 (15.34-27.72) | **-24.87 (-38.18, -8.70)** |  | -8.70 (-41.90, 43.62) |
| 4 (>27.72) | **-27.02 (-40.73, -10.15)** |  | 12.19 (-31.13, 82.94) |
| ∑DEHP^2^ |  |  |  |
| 1^5^ (<0.10) | Ref |  | Ref |
| 2 (0.10-0.19) | **-18.21 (-32.23, -1.39)** |  | 13.88 (-24.57, 71.94) |
| 3 (0.19-0.35) | **-19.75 (-33.83, -2.66)** |  | 2.22 (-35.73, 62.74) |
| 4 (>0.35) | **-22.66 (-37.19, -4.78)*** |  | 19.12 (-25.32, 90.03) |
| MOP^3^ | -5.16 (-17.30, 8.76) |  | 1.92 (-26.07, 40.49) |
| *Tests for linear trend with *P*-value < 0.05. Statistically significant results comparing a specific category to the reference are bolded. | | | |
| Models were adjusted for age, BMI and creatinine. | | | |
| ^1^Serum INHB levels were natural logarithm transformed. | | | |
| ^2^Phthalate metabolite concentrations were categorized into quartiles. | | | |
| ^3^Dichotomous variable based on above/below limits of detection. | | | |
| ^4^Percent change and 95% CI were calculated as follows: [exp (β)-1]*100.  ^5^Reference category. | | | |
|  | | | |
