# Supplementary material for: Urinary phthalate metabolites in relation to serum anti-Müllerian hormone and inhibin B levels among women from a fertility center: a retrospective analysis
Source: Reprod Health. 2018 Feb 23;15:33. doi: 10.1186/s12978-018-0469-8 (PMC5824533; doi:10.1186/s12978-018-0469-8)
Supplement: Supplementary file 4 — Adjusted odds ratios (95% CI) for polycystic ovarian morphology (PCOM) and diminished ovarian reserve (DOR) by urinary phthalate metabolites (n = 415). (DOCX 15 kb) [file 12978_2018_469_MOESM4_ESM.docx]

| **Table S3 Adjusted odds ratios (95% CI) for polycystic ovarian morphology (PCOM) and diminished ovarian reserve (DOR) by urinary phthalate metabolites (n=415).** | | | |
| --- | --- | --- | --- |
| Metabolite | PCOM^1^ |  | DOR^2^ |
|  | OR (95% CI) |  | OR (95% CI) |
| MMP^3^ |  |  |  |
| 1^5^ (<5.18) | Ref |  | Ref |
| 2 (5.18-12.21) | 0.69 (0.37, 1.30) |  | 1.51 (0.57, 3.95) |
| 3 (12.21-25.78) | 0.60 (0.32, 1.14) |  | 1.17 (0.40, 3.39) |
| 4 (>25.78) | 0.93 (0.49, 1.74) |  | 1.41 (0.52, 3.83) |
| MEP^3^ |  |  |  |
| 1^5^ (<6.02) | Ref |  | Ref |
| 2 (6.02-12.80) | 0.64 (0.34, 1.23) |  | 1.46 (0.52, 4.09) |
| 3 (12.80-33.98) | 0.82 (0.42, 1.59) |  | 1.26 (0.46, 3.45) |
| 4 (>33.98) | 0.82 (0.42, 1.59) |  | 1.32 (0.47, 3.68) |
| MBP^3^ |  |  |  |
| 1^5^ (<73.85) | Ref |  | Ref |
| 2 (73.85-184.55) | **2.15 (1.13, 4.09)** |  | 0.38 (0.13, 1.13) |
| 3 (184.55-342.12) | 0.94 (0.47, 1.87) |  | 0.86 (0.32, 2.32) |
| 4 (>342.12) | 1.09 (0.50, 2.39) |  | 0.50 (0.16, 1.54) |
| MBzP^3^ |  |  |  |
| 1^5^ (<0.035) | Ref |  | Ref |
| 2 (0.035-0.102) | 0.56 (0.30, 1.06) |  | 0.84 (0.31, 2.30) |
| 3 (0.102-0.27) | 0.76 (0.38, 1.49) |  | 1.22 (0.45, 3.32) |
| 4 (>0.27) | 1.18 (0.61, 2.30) |  | 0.64 (0.22, 1.92) |
| MEHP^3^ |  |  |  |
| 1^5^ (<6.95) | Ref |  | Ref |
| 2 (6.95-17.21) | 1.62 (0.86, 3.04) |  | 0.60 (0.24, 1.54) |
| 3 (17.21-36.01) | 0.93 (0.48, 1.82) |  | 0.66 (0.25, 1.74) |
| 4 (>36.01) | **2.58 (1.29, 5.16)*** |  | 0.48 (0.17, 1.31) |
| MEHHP^3^ |  |  |  |
| 1^5^ (<10.94) | Ref |  | Ref |
| 2 (10.94-19.09) | 1.00 (0.52, 1.91) |  | 0.82 (0.31, 2.18) |
| 3 (19.09-34.68) | 1.03 (0.50, 2.12) |  | 0.57 (0.19, 1.72) |
| 4 (>34.68) | 1.75 (0.83, 3.69) |  | 0.52 (0.16, 1.63) |
| MEOHP^3^ |  |  |  |
| 1^5^ (<7.41) | Ref |  | Ref |
| 2 (7.41-15.34) | 1.03 (0.54, 1.96) |  | 0.89 (0.32, 2.44) |
| 3 (15.34-27.72) | 0.95 (0.47, 1.89) |  | 0.96 (0.35, 2.68) |
| 4 (>27.72) | 1.84 (0.88, 3.82) |  | 0.73 (0.23, 2.26) |
| ∑DEHP^3^ |  |  |  |
| 1^5^ (<0.10) | Ref |  | Ref |
| 2 (0.10-0.19) | 0.98 (0.51, 1.87) |  | 0.62 (0.24, 1.65) |
| 3 (0.19-0.35) | 0.73 (0.37, 1.45) |  | 0.56 (0.20, 1.57) |
| 4 (>0.35) | 1.84 (0.89, 3.79) |  | 0.54 (0.19, 1.57) |
| MOP^4^ | 1.50 (0.93, 2.41) |  | 0.49 (0.21, 1.13) |
| *Tests for linear trend with *P*-value < 0.05. Statistically significant results comparing a specific category to the reference are bolded. | | | |
| Models were adjusted for age, BMI and creatinine. | | | |
| ^1^PCOM was defined as AMH>5ng/mL vs. “normal” (AMH≤5 ng/mL). | | | |
| ^2^DOR was defined as AMH<1.1 ng/mL vs. “normal” (AMH≥1.1 ng/mL). | | | |
| ^3^Phthalate metabolite concentrations were categorized into quartiles. | | | |
| ^4^Dichotomous variable based on above/below limits of detection. | | | |
| ^5^Reference category. | | | |
