# Supplementary material for: Urinary phthalate metabolites in relation to serum anti-Müllerian hormone and inhibin B levels among women from a fertility center: a retrospective analysis
Source: Reprod Health. 2018 Feb 23;15:33. doi: 10.1186/s12978-018-0469-8 (PMC5824533; doi:10.1186/s12978-018-0469-8)
Supplement: Supplementary file 5 — Associations between urinary phthalate metabolites and AFC in multivariable generalized linear models (n = 415). (DOCX 14 kb) [file 12978_2018_469_MOESM5_ESM.docx]

| **Table S4 Associations between urinary phthalate metabolites and AFC in multivariable generalized linear models (n=415).** | | | |
| --- | --- | --- | --- |
| Metabolite | Model 1^3^ |  | Model 2^4^ |
|  | β (95% CI) |  | β (95% CI) |
| MMP^1^ |  |  |  |
| 1^5^ (<5.18) | Ref |  | Ref |
| 2 (5.18-12.21) | **−0.79 (−0.16, −0.002)** |  | 0.001 (−0.08, 0.08) |
| 3 (12.21-25.78) | −0.06 (−0.14, 0.02) |  | 0.07 (−0.01, 0.15) |
| 4 (>25.78) | −0.01 (−0.08, 0.02) |  | 0.01 (−0.07, 0.09) |
| MEP^1^ |  |  |  |
| 1^5^ (<6.02) | Ref |  | Ref |
| 2 (6.02-12.80) | −0.05 (−0.13, 0.03) |  | −0.03 (−0.11, 0.05) |
| 3 (12.80-33.98) | 0.03 (−0.06, 0.11) |  | −0.01 (−0.09, 0.07) |
| 4 (>33.98) | −0.01 (−0.09, 0.08) |  | −0.03 (−0.11, 0.05) |
| MBP^1^ |  |  |  |
| 1^5^ (<73.85) | Ref |  | Ref |
| 2 (73.85-184.55) | **0.16 (0.08, 0.24)** |  | 0.08 (−0.004, 0.16) |
| 3 (184.55-342.12) | 0.05 (−0.04, 0.14) |  | −0.02 (−0.11, 0.07) |
| 4 (>342.12) | **0.17 (0.07, 0.26)** |  | **0.11 (0.01, 0.20)** |
| MBzP^1^ |  |  |  |
| 1^5^ (<0.035) | Ref |  | Ref |
| 2 (0.035-0.102) | −0.01 (−0.09, 0.07) |  | −0.003 (−0.08, 0.07) |
| 3 (0.102-0.27) | −0.03 (−0.12, 0.05) |  | 0.04 (−0.05, 0.12) |
| 4 (>0.27) | 0.01 (−0.07, 0.09) |  | 0.03 (−0.05, 0.11) |
| MEHP^1^ |  |  |  |
| 1^5^ (<6.95) | Ref |  | Ref |
| 2 (6.95-17.21) | 0.05 (−0.03, 0.13) |  | 0.04 (−0.04, 0.11) |
| 3 (17.21-36.01) | 0.02 (−0.06, 0.10) |  | −0.02 (−0.10, 0.06) |
| 4 (>36.01) | 0.04 (−0.05, 0.12) |  | 0.01 (−0.08, 0.09) |
| MEHHP^1^ |  |  |  |
| 1^5^ (<10.94) | Ref |  | Ref |
| 2 (10.94-19.09) | 0.02 (−0.06, 0.10) |  | 0.03 (−0.05, 0.11) |
| 3 (19.09-34.68) | 0.05 (−0.04, 0.14) |  | 0.05 (−0.04, 0.14) |
| 4 (>34.68) | **0.14 (0.05, 0.23)*** |  | 0.09 (−0.01, 0.18) |
| MEOHP^1^ |  |  |  |
| 1^5^ (<7.41) | Ref |  | Ref |
| 2 (7.41-15.34) | 0.01 (−0.07, 0.09) |  | −0.03 (−0.11, 0.05) |
| 3 (15.34-27.72) | −0.01 (−0.10, 0.08) |  | −0.02 (−0.11, 0.06) |
| 4 (>27.72) | **0.14 (0.05, 0.23)*** |  | 0.04 (−0.05, 0.13) |
| ∑DEHP^1^ |  |  |  |
| 1^5^ (<0.10) | Ref |  | Ref |
| 2 (0.10-0.19) | **0.11 (0.03, 0.19)** |  | 0.05 (−0.03, 0.13) |
| 3 (0.19-0.35) | −0.004 (−0.09, 0.08) |  | −0.02 (−0.11, 0.06) |
| 4 (>0.35) | **0.12 (0.03, 0.21)** |  | 0.06 (−0.03, 0.14) |
| MOP^2^ | 0.02 (−0.04, 0.08) |  | 0.05 (−0.01, 0.11) |
| *Tests for linear trend with *P*-value < 0.05. Statistically significant results comparing a specific category to the reference are bolded. | | | |
| ^1^Phthalate metabolite concentrations were categorized into quartiles. | | | |
| ^2^Dichotomous variable based on above/below limits of detection. | | | |
| ^3^Model 1 was adjusted for age, BMI and creatinine. | | | |
| ^4^Model 2 was adjusted for age, BMI, creatinine and PCO/PCOS diagnosis (yes or no). | | | |
| ^5^Reference category. | | | |
